# Progress test performance of medical students admitted via the medical education preparation program

**DOI:** 10.3389/fmed.2026.1737581

**Published:** 2026-01-21

**Authors:** Gita Sekar Prihanti, Naila Fairuz Salmaa

**Affiliations:** Department of Medical Education, Faculty of Medicine, Universitas Muhammadiyah Malang, Malang, Indonesia

**Keywords:** widening participation, Progress test, medical students, academic achievement, clinical rotation stage, medical education preparation program

## Abstract

The progress test is a periodic evaluation designed to assess the development of medical students’ knowledge during the professional stage. The Medical Education Preparation Program (Program Persiapan Pendidikan Dokter/P3D) was established as part of a widening participation initiative to expand inclusive access to medical education. This study aimed to analyze differences in progress test scores between P3D and regular students at the Faculty of Medicine, Universitas Muhammadiyah Malang. An analytical observational study with a retrospective cohort design was conducted using secondary data from progress test scores collected between 2022 and 2024. A total of 116 students were selected through random sampling, and the data were analyzed using independent t-tests and linear regression. The results showed no significant difference in progress test scores between P3D and regular students (*p* = 0.891). However, a significant difference was observed in the 2016 cohort (*p* = 0.004), with P3D students achieving higher mean scores. A decline in performance among more recent cohorts may have been influenced by external factors such as the COVID-19 pandemic. In conclusion, the academic performance of P3D and regular students was generally equivalent, indicating that the program effectively prepares students for the clinical rotation stage. These findings support the continuation of P3D as an inclusive admission pathway to strengthen the quality of medical education.

## 1 Introduction

Diversity among medical students is a key factor in enhancing educational quality and ensuring equitable access to high-standard healthcare services. However, admission to medical school is often regarded as highly competitive, emotionally demanding, and offering limited opportunities for applicants from underrepresented groups (1). Such inequality may hinder access for individuals with strong academic potential who are disadvantaged by socioeconomic status, rural origin, first-generation higher education background, or disability.

To address these challenges, many higher education institutions have implemented widening participation (WP) initiatives aimed at broadening access, increasing participation, and supporting academic success among students from underrepresented backgrounds (2–4). Several universities worldwide have developed WP programs, including the Gateway to Medicine Programme in the United Kingdom, which provides a one-year academic preparation consisting of tutorials, simulations, and skills training to facilitate the transition into medical education (5).

In Indonesia, the Faculty of Medicine at Universitas Muhammadiyah Malang (FK UMM) has introduced the Medical Education Preparation Program (Program Persiapan Pendidikan Dokter/P3D) as a local adaptation of WP. This program offers one year of academic and non-academic preparation to help students adapt to the demanding medical curriculum. The initiative is expected to bridge access gaps, particularly for applicants from diverse educational backgrounds, while maintaining the quality of graduates.

Evaluating the effectiveness of WP programs requires robust and continuous assessment tools. One widely adopted instrument is the progress test, a longitudinal formative assessment designed to measure the cumulative knowledge of medical students throughout their training (6, 7). The progress test not only reflects individual academic performance but also serves as an indicator for comparing outcomes among students admitted through different pathways.

Although WP has been shown to improve access and diversity, empirical evidence on the academic performance of students admitted through such pathways remains limited, particularly in Indonesia. It is therefore important to determine whether students admitted through P3D achieve academic outcomes comparable to those of their peers.

This study analyzes differences in progress test performance between P3D and regular students at FK UMM during the professional stage, providing insights into the effectiveness of P3D as an inclusive pathway in medical education.

## 2 Materials and methods

This study employed an observational analytic design with a retrospective cohort approach, using secondary data from progress test scores of professional-stage medical students at the Faculty of Medicine, Universitas Muhammadiyah Malang (UMM). The dataset consisted of progress test results collected between 2022 and 2024 from students admitted between 2016 and 2021.

The study population included all professional-stage students from both the Medical Education Preparation Program (P3D) and the regular admission pathway. A total of 116 students were selected through random sampling with equal representation from both groups. The research instrument comprised secondary data on progress test scores ranging from 0 to 100, obtained from the official archives of the academic office, representing the cumulative assessment of students’ knowledge during the professional stage.

Data analysis was performed using statistical software. Descriptive statistics were used to describe respondent characteristics and score distribution, while independent t-tests were applied to compare mean scores between P3D and regular students. Linear regression analysis was further conducted to assess the influence of admission pathway and other variables on academic performance, with statistical significance set at *p* < 0.05.

Ethical approval for this study was granted by the Health Research Ethics Committee of the Faculty of Medicine, Universitas Muhammadiyah Malang (No. E.5.a/083/KEPKUMM/V/2025). All data were anonymized prior to analysis to ensure participant confidentiality.

## 3 Results

A total of 116 professional-stage students were included in the analysis, comprising 58 from the P3D pathway and 58 from the regular pathway. Female students predominated (59.5%) compared to males (40.5%), with the largest proportion originating from the 2016 cohort (22.4%) (Table 1).

**Table 1:** Characteristics of study respondents.

Category	Description	P3D *N* (%)	Regular *N* (%)
Total		58 (50%)	58 (50%)
Gender			
Male		25 (21.6%)	22 (19.0%)
Female		33 (28.4%)	36 (31.0%)
Cohort	Year of progress test		
2016	2022	13 (11.2%)	13 (11.2%)
2017	2023	11 (9.5%)	11 (9.5%)
2018	2024	7 (6.0%)	7 (6.0%)
2019	2024	11 (9.5%)	11 (9.5%)
2020	2023	6 (5.2%)	6 (5.2%)
2021	2024	10 (8.6%)	10 (8.6%)
		Mean ± SD (P3D)	Mean ± SD (Regular)
Progress test		40.32 ± 10.32	40.55 ± 7.79

The mean progress test score of P3D students was 40.32 ± 10.32, compared with 40.55 ± 7.79 for regular students. The difference of 0.23 points was not statistically significant (*p* = 0.891). The independent t-test also revealed no significant difference between male and female students (*p* = 0.210).

Cohort-based analysis showed that only the 2016 cohort demonstrated a significant difference (*p* = 0.004), with P3D students achieving higher mean scores than regular students, while no meaningful differences were observed in other cohorts. Overall, earlier cohorts (2016, 2017, 2018) tended to have higher average progress test scores compared with more recent cohorts (2019, 2020, 2021). The 2020 and 2021 cohorts recorded lower performance relative to the others (Table 2).

**Table 2:** Comparison of progress test mean scores by admission track, gender, and cohort.

Category	Description	P3D N (%)	Regular *N* (%)	Total *N* (%)	*p*-value*
Gender					
Male		25 (21.6%)	22 (19.0%)	47 (40.5%)	0.270
Female		33 (28.4%)	36 (31.0%)	69 (59.5%)	0.463
Cohort	Semester of progress test				
2016	4 (clinical stage)	13 (11.2%)	13 (11.2%)	26 (22.4%)	0.004
2017	4 (clinical stage)	11 (9.5%)	11 (9.5%)	22 (19.0%)	0.456
2018	4 (clinical stage)	7 (6.0%)	7 (6.0%)	14 (12.1%)	0.773
2019	4 (clinical stage)	11 (9.5%)	11 (9.5%)	22 (19.0%)	0.255
2020	7 (pre-clinical stage)	6 (5.2%)	6 (5.2%)	12 (10.3%)	0.206
2021	7 (pre-clinical stage)	10 (8.6%)	10 (8.6%)	20 (17.2%)	0.960
		Mean ± SD (P3D)	Mean ± SD (Regular)	Mean ± SD (Total)	
Progress test		40.32 ± 10.32	40.55 ± 7.79	40.44 ± 9.11	0.891

*Independent *T*-test.

Further linear regression analysis indicated that neither gender (*p* = 0.900) nor admission pathway (*p* = 0.836) significantly influenced progress test performance. The regression model explained only 0.1% of score variation, suggesting that other factors likely play a greater role in students’ academic achievement (Table 3).

**Table 3:** Regression analysis of the effect of gender and admission track on progress test scores.

Variable	*B*	*P*-value*	*R* square
Intercept	39.540		0.001
Gender	0.214	0.900	
Admission track	0.362	0.836	

*Linear regression.

## 4 Discussion

This study showed that the progress test scores of P3D students did not differ significantly from those of non-P3D students, indicating that both groups achieved comparable academic outcomes. The small mean difference suggests that the academic potential of P3D students is equivalent to that of students admitted through the regular pathway.

These findings align with previous studies comparing the performance of pre-medical and regular medical track students, which also reported no significant differences in academic achievement (8). Similarly, a recent study found no significant differences in Annual Review of Competence Progression (ARCP) outcomes between graduates of gateway courses and those from standard entry medicine, suggesting comparable performance across pathways (9). This reinforces the view that alternative admission routes, such as the P3D program, can produce graduates with competencies equivalent to those admitted through conventional entry.

Nonetheless, other studies have reported contrasting results, indicating that admission mechanisms may influence long-term academic performance (10) and that the type of entry pathway can be associated with cumulative grade point average outcomes (11). Such variations highlight that the effectiveness of alternative admission routes is highly dependent on institutional context and program design.

Analysis by entry cohort revealed a significant difference in the 2016 intake, where P3D students achieved higher scores than their non-P3D counterparts. This may be attributed to stronger learning motivation and better time management skills (12), as well as longer study experience that supports examination readiness (13). Overall, earlier cohorts (2016–2018) recorded higher mean progress test scores compared with more recent cohorts (2019–2021). This trend is consistent with previous findings showing that senior students tend to perform better academically as their knowledge and experience accumulate over time (7, 13).

Cohorts entering in 2020 and 2021 demonstrated lower overall performance, likely reflecting the impact of the COVID-19 pandemic, which diminished the effectiveness of higher education learning (14). Gender was not significantly associated with progress test outcomes, aligning with evidence that academic achievement is generally not influenced by gender differences (15). Instead, non-biological factors such as motivation (16), time management (17), family support (18), and peer influence (19) are more likely to play a decisive role, as highlighted in previous studies on predictors of academic performance.

These findings have important implications for the sustainability of the P3D program. Beyond functioning as a selection mechanism, P3D serves as a one-year academic and non-academic preparation system that enables students to develop skills and readiness for the professional phase. This concept aligns with the Gateway to Medicine Programme in the United Kingdom, which broadens access to medical education without compromising graduate quality (20), as well as with similar preparatory initiatives in other countries that have been shown to enhance student readiness for the professional stage (21).

At the Faculty of Medicine, Universitas Muhammadiyah Malang, P3D is implemented through administrative selection, two semesters of structured coursework, and phased evaluation with clear academic criteria (22). The program’s success is further supported by active student engagement, particularly in developing self-directed learning, motivation, communication (23), and critical thinking skills (24). Moreover, the integration of active learning strategies—such as motivational interviewing, think-aloud, retrieval practice, and peer teaching—together with senior student mentoring, has contributed to improved academic outcomes (25, 26). Therefore, P3D can be considered an effective widening participation model with potential for adoption by other medical schools in Indonesia.

This study has several limitations. Its scope was restricted to a single institution with limited variables, namely preparatory program type and gender. Other non-biological factors, such as motivation, learning strategies, and social support, were not directly analyzed, although they may influence learning outcomes and should be considered in future research. The data relied on academic records, which depended on the completeness of documentation and may have introduced bias. In addition, the progress test primarily assessed cognitive aspects and may not fully capture students’ clinical skills.

To address these limitations, future studies should adopt a prospective multicenter design with a broader range of variables and evaluate the effectiveness of P3D not only through progress test results but also with additional indicators such as Grade Point Average (GPA) and clinical readiness.

## 5 Conclusion

P3D students demonstrated progress test performance comparable to that of students admitted through the regular pathway at the professional stage. A significant difference was observed only in the 2016 cohort, where P3D students achieved higher scores. Neither admission pathway nor gender influenced performance, indicating that entry route was not a major determinant of academic outcomes. These findings suggest that P3D is an effective alternative admission pathway that can be further developed to expand access to medical education without compromising academic quality.

## Data Availability

The original contributions presented in the study are included in the article/supplementary material, further inquiries can be directed to the corresponding author.
